# Triple modal treatment comprising with proton beam radiation, hyperthermia, and gemcitabine/nab-paclitaxel for locally advanced pancreatic cancer: a phase I/II study protocol (TT-LAP trial)

**DOI:** 10.1186/s12885-023-11110-y

**Published:** 2023-07-04

**Authors:** Osamu Shimomura, Masato Endo, Hirokazu Makishima, Takeshi Yamada, Shinji Hashimoto, Haruko Numajiri, Yoshihiro Miyazaki, Manami Doi, Kinji Furuya, Kazuhiro Takahashi, Toshikazu Moriwaki, Naoyuki Hasegawa, Yoshiyuki Yamamoto, Yusuke Niisato, Mariko Kobayashi, Masashi Mizumoto, Kei Nakai, Takashi Saito, Sodai Hoshiai, Tsukasa Saida, Bryan J. Mathis, Kensaku Mori, Takahito Nakajima, Kiichiro Tsuchiya, Hideyuki Sakurai, Tatsuya Oda

**Affiliations:** 1 Department of Gastrointestinal and Hepato-Biliary-Pancreatic Surgery, Faculty of Medicine, University of Tsukuba, Ibaraki, 305-8575 Japan; 2 Department of Gastroenterology, University of Tsukuba, Ibaraki, Japan; 3 Department of Radiation oncology, University of Tsukuba, Ibaraki, Japan; 4 Department of Radiology, University of Tsukuba, Ibaraki, Japan; 5 International Medical Center, University of Tsukuba Hospital, Ibaraki, Japan

**Keywords:** Locally advanced pancreatic cancer, Clinical trial, Multidisciplinary treatment, Proton beam therapy, Hyperthermia

## Abstract

**Background:**

Locally advanced pancreatic ductal adenocarcinoma (PDAC), accounting for about 30% of PDAC patients, is difficult to cure by radical resection or systemic chemotherapy alone. A multidisciplinary strategy is required and our TT-LAP trial aims to evaluate whether triple-modal treatment with proton beam therapy (PBT), hyperthermia, and gemcitabine plus nab-paclitaxel is a safe and synergistically effective treatment for patients with locally advanced PDAC.

**Methods:**

This trial is an interventional, open-label, non-randomized, single-center, single-arm phase I/II clinical trial organized and sponsored by the University of Tsukuba. Eligible patients who are diagnosed with locally advanced pancreatic cancer, including both borderline resectable (BR) and unresectable locally advanced (UR-LA) patients, and selected according to the inclusion and exclusion criteria will receive triple-modal treatment consisting of chemotherapy, hyperthermia, and proton beam radiation. Treatment induction will include 2 cycles of chemotherapy (gemcitabine plus nab-paclitaxel), proton beam therapy, and 6 total sessions of hyperthermia therapy. The initial 5 patients will move to phase II after adverse events are verified by a monitoring committee and safety is ensured. The primary endpoint is 2-year survival rate while secondary endpoints include adverse event rate, treatment completion rate, response rate, progression-free survival, overall survival, resection rate, pathologic response rate, and R0 (no pathologic cancer remnants) rate. The target sample size is set at 30 cases.

**Discussion:**

The TT-LAP trial is the first to evaluate the safety and effectiveness (phases1/2) of triple-modal treatment comprised of proton beam therapy, hyperthermia, and gemcitabine/nab-paclitaxel for locally advanced pancreatic cancer.

**Ethics and dissemination:**

This protocol was approved by the Tsukuba University Clinical Research Review Board (reference number TCRB22-007). Results will be analyzed after study recruitment and follow-up are completed. Results will be presented at international meetings of interest in pancreatic cancer plus gastrointestinal, hepatobiliary, and pancreatic surgeries and published in peer-reviewed journals.

**Trial registration:**

Japan Registry of Clinical Trials, jRCTs031220160. Registered 24 th June 2022, https://jrct.niph.go.jp/en-latest-detail/jRCTs031220160.

## Introduction

Pancreatic ductal adenocarcinoma (PDAC), due to its aggressive invasion of the nearby arterial plexus and frequent metastasis, ranks as the 14^th^ most common cancer but carries the worst 5-year overall survival (OS) rate (9% among all solid cancer types) [1]. It is also estimated to become the second-leading cause of cancer death by 2030 [2]. Because of frequent late-stage diagnosis, roughly 50% of all PDAC patients already have distant metastasis at the time of initial diagnosis (unresectable distant metastasis; UR-M). In these cases, systemic chemotherapy is considered mandatory; however, best-effort median survival times of less than 1 year frustrate attempts to control progression with chemotherapy alone [3]. Additionally, up to 30% of patients are diagnosed with locally advanced pancreatic cancer (LAPC), including borderline resectable (BR) and unresectable locally advanced (UR-LA) types, as defined by vascular invasion status. Although multidisciplinary treatment in these cases, centered on surgical resection, is essential to achieve a cure, candidates for resection at this stage are limited. Moreover, high recurrence rates after radical resection are frequently observed as these patients, especially those with positive arterial plexus invasion, cannot achieve pathologically complete resection (R0 resection) without pre-operative treatment. Thus, a majority of advanced PDAC patients can be expected to progress to systemic chemotherapy.

While diverse clinical trials have reported treatment strategies for LAPC, a gold standard regimen remains to be established as multidisciplinary chemo/radiotherapy combinations have failed to improve 5-year OS rates. This was seen in previously reported chemoradiation therapy studies where overall survival did not change but progression-free survival did improve [4]. Thus, many trials rely on systemic chemotherapy under the reasonable assumption that micro-metastases are systemically distributed in locally advanced PDAC. However, such reported single-agent, systemic chemotherapy regimens might be less effective. Furthermore, the efficacy of chemoradiation therapy is unclear when combined with recently verified regimens that attack tumor DNA synthesis on multiple fronts, such as FOLFIRINOX (folinic acid, 5-fluorouracil, irinotecan, and oxaliplatin) or gemcitabine (a known radiosensitizer) plus nab-paclitaxel [3, 5, 6]. These synergistic combinations of DNA synthesis/cell division inhibitors can be exploited to quickly suppress metastasis and enhance radiation lethality before resistance occurs. In a 2013 study, gemcitabine plus nab-paclitaxel alone offered a 1.8-month (~21%) improvement (8.5 months) compared to gemcitabine alone (6.7 months) in median overall survival for metastatic pancreatic cancer [6]. A meta-analysis of 11 reports totaling 315 LAPC patients found that FOLFIRINOX treatment had median overall survival times of 24.2 months when combined with adjuvant radiotherapy and surgical resection [7]. Such multi-pronged regimens featuring these therapeutics may therefore be useful in LAPC to suppress metastasis, reduce resistance due to possible cancer stem cell populations, and increase both progression-free and overall survival [8].

We recently published a retrospective study about the feasibility and effectiveness of a triple-modal strategy that combines gemcitabine-based chemotherapy, proton beam therapy (PBT), and local hyperthermia for UR-LA patients with major-artery invasion [9]. In this report, 21 UR-LA patients achieved a median 28.0 months of overall survival while the survival duration of those who underwent conversion surgery after triple modality treatment did not change significantly compared to controls. We chose gemcitabine as the core chemotherapy component in our regimen. We also discovered that PBT was superior to photon radiation for survival and our triple-modal therapy resulted in cases with a strong local effect that resulted in pathologic complete response (pCR). However, many patients experienced distant metastasis after triple-modal treatment, indicating a need for more intense systemic treatment from the beginning. The third part of the triple-modal treatment features hyperthermia, which has been demonstrated to damage tumor DNA, destabilize oncogene products through heat shock protein upregulation, and induce immunogenic cell death [10]. A recent clinical trial [11] used hyperthermia, gemcitabine, and radiation but has yet to post results. Thus, we aim to evaluate the safety and the efficacy of our triple modal treatment featuring PBT, hyperthermia, and gemcitabine/nab-paclitaxel in BR-A and UR, locally advanced pancreatic cancer.

## Methods and study design

This trial is an interventional, open-label, non-randomized, single-center, single-arm phase I and II clinical trial organized and sponsored by the University of Tsukuba. This single-center study will be conducted at the University of Tsukuba Hospital. Eligible patients who are diagnosed with locally advanced pancreatic cancer and selected according to the inclusion and exclusion criteria listed below will receive triple-modal treatment, including PBT, hyperthermia, and chemotherapy.

### Objectives and endpoints

#### Objectives

The aim of this trial is to assess the safety of the triple-modal treatment comprised of PBT, hyperthermia, and gemcitabine with nab-paclitaxel as the phase I part and the efficacy and safety of this strategy as the phase II part.

#### Endpoints

The primary outcome for phase I is drug-limiting toxicity (DLT) and the 2-year survival rate is the phase II primary outcome. The primary and the secondary outcomes for each phase are summarized in Table 1.

**Table 1:**
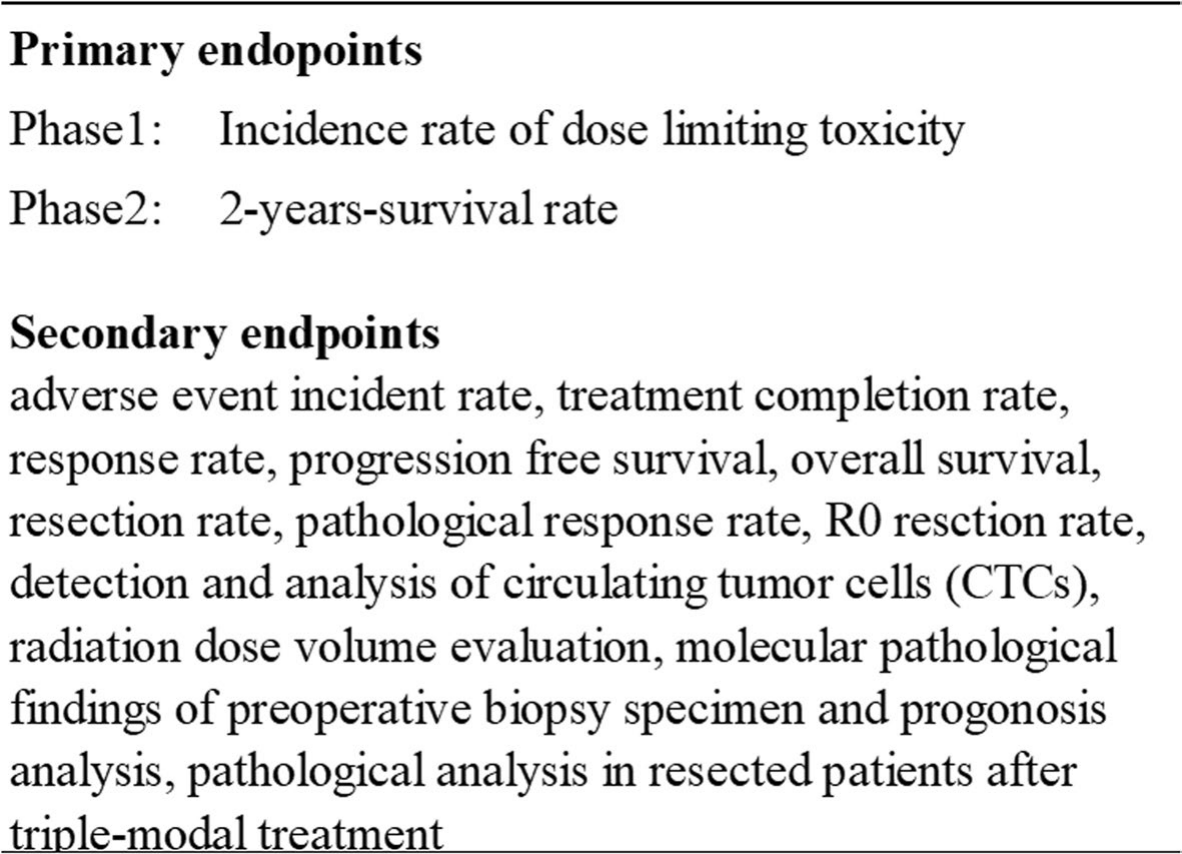
Endpoints of the TT-LAP trial

### Patient registration

This trial includes patients who are diagnosed with locally advanced pancreatic cancer, including both borderline resectable with arterial invasion (BR-A) and unresectable locally advanced (UR-LA), according to National Comprehensive Cancer Network (NCCN) guidelines Version 1.2021. The diagnosis of primary tumors will be performed by thin-slice (slice thickness of 2.5 mm or less) dynamic CT and board-certified radiologists, surgeons, physicians and radiation oncologists in clinical concert at the Tsukuba Pancreato-Biliary Tumor Center. Patients with distant organ metastases, including para-aortic lymph node metastasis and/or peritoneal dissemination, will be excluded from this trial. The evaluation of distant metastases will be conducted by lung CT scans and/or liver MRI with gadoxetate disodium as contrast agent (e.g., Primovist™), with FDG-PET (18F-fluorodeoxyglycose positron emission tomography) considered if the diagnosis of distant metastases is difficult. Eligible patients will be screened according to the following criteria:

Inclusion criteria:

A) Histological or cytological diagnosis of pancreatic ductal adenocarcinoma (PDAC).
B) Radiologically diagnosed as BR (borderline resectable) or UR-LA (unresectable locally advanced) according to NCCN guidelines.
C) Performance Status (PS): 0-1 (ECOG criteria).
D) Patients with normal major organ function (heart, kidneys, liver, bone marrow, lungs, brain, etc.) reflected in the following lab values: hemoglobin ≥ 9.0g/dl, neutrocyte ≥ 1,500/mm^3^, platelet ≥ 75,000/mm^3^, total bilirubin ≤ 3.0mg/dl, AST ≤ ULN x 5, ALT ≤ ULN x 5, and creatinine clearance > 40 mL/min (according to the Cockroft-Gult formula)
E) Between ages 20 and 80.
F) Patients who can receive proton beam therapy.
G) Patients who give written, informed consent.

Exclusion criteria:

A) Patients with distant metastases, including para-aortic lymph node metastasis and/or peritoneal dissemination.
B) Patients with severe drug-induced hypersensitivity syndrome.
C) Patients with other, concurrent invasive cancers with less than 5 years of expected disease-free survival.
D) Patients with allergies for contrast materials and difficult-to-diagnose pancreatic ductal adenocarcinomas.
E) Past treatment history for pancreatic cancer and resistance for any treatments
F) Patients who received dose overlap with previous irradiation or for whom this cannot be ruled out.
G) Patients with comorbidities that carry contraindications to radiation therapy (active collagen disease, xeroderma pigmentosum, etc.).
H) Patients with clear endoscopic exposure of cancer in the mucosa of the gastrointestinal tract.
I) Patients with HCV RNA virus-positive or HBs antigen-positive hepatitis statuses.
J) Patients on dialysis with chronic renal failure or Child-Pugh B or higher-class liver cirrhosis.
K) Patients who are, may be, or wish to become pregnant plus those who are breastfeeding.
L) Patients with severe mental disabilities.

### Treatment protocol

**Fig. 1:**
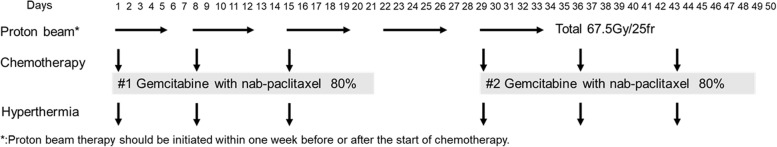
The flowchart of the TT-LAP trial. BR-A, borderline resectable with artery invasion; UR-LA, unresectable locally advanced; IDMC, independent data monitoring committee. ※If less than 1 case of DLT occurs among 5 patients and safety is confirmed, proceed to phase 2

This is a phase I/II study to evaluate the safety and efficacy of gemcitabine/nab-paclitaxel therapy in combination with PBT and local hyperthermia in patients with locally advanced pancreatic cancer (Fig. 1). Two rounds of gemcitabine/nab-paclitaxel therapy will be administered on a 4-week cycle (3-week dosing, 1-week rest), which is the recommended schedule for pancreatic cancer according to 2019 clinical practice guidelines from the Japan Pancreas Society (JPS), and hyperthermia will be administered for 1 hour on the same day as chemotherapy. PBT will be initiated within 1 week before or after the start of chemotherapy. The prescribed PBT dose will be set to 67.5 Gy in 27 fractions. Hyperthermia should be started within 4 hours after completion of chemotherapy in order to achieve a synergistic effect with chemotherapy but a 1-hour buffer will be allowed depending on the availability of equipment. Treatment protocols done after this study period will not be analyzed as part of this trial. Appropriate treatment will be provided under the direction of specialists according to the guidelines for pancreatic cancer.

### Safety evaluations for phase I

**Fig. 2:**
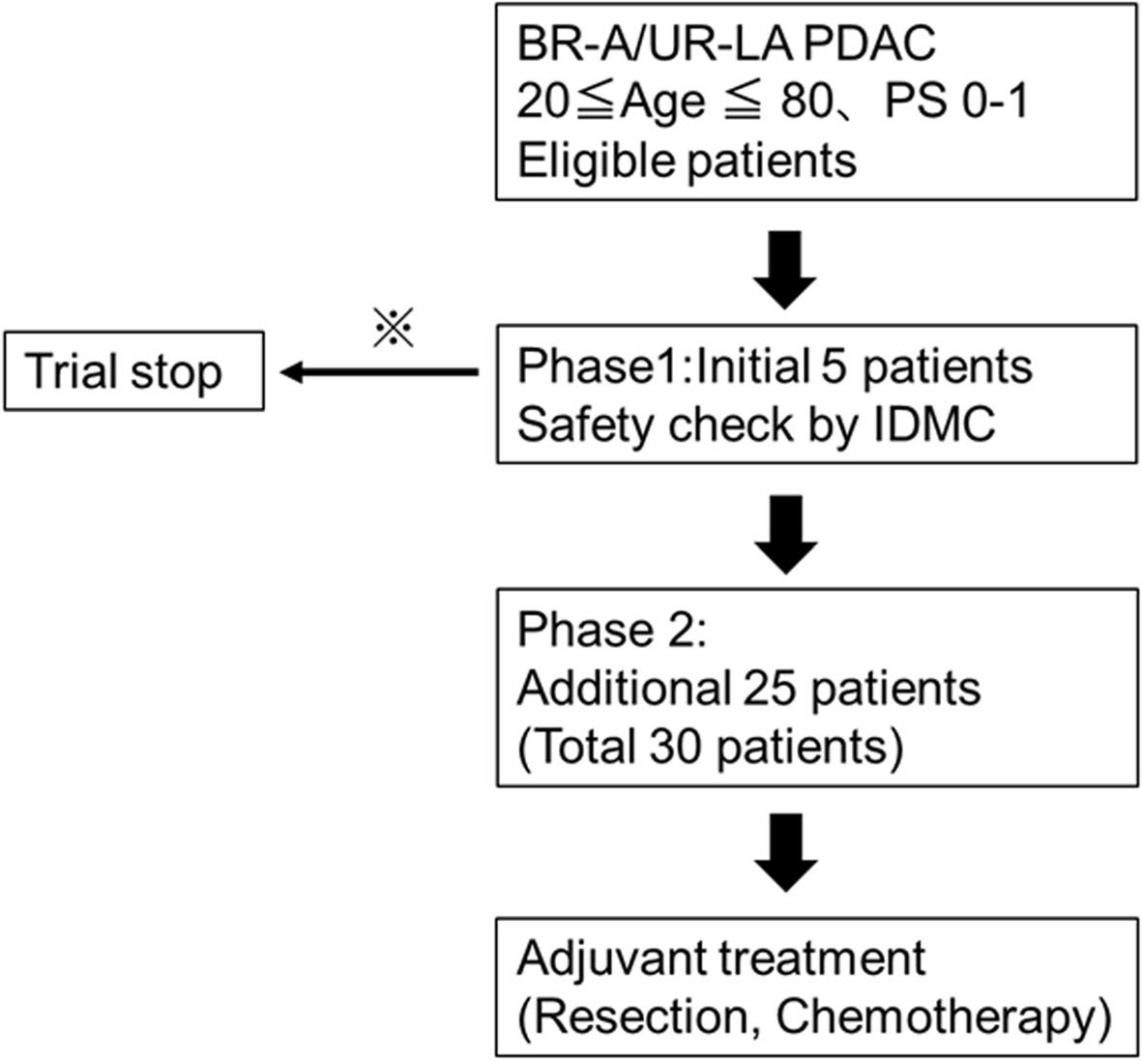
Treatment schedule of the TT-LAP trial. The TT-LAP trial tests a triple-modal treatment, consisting of proton beam therapy, gemcitabine/nab-paclitaxel, and hyperthermia. Chemotherapy will be administered for 2 cycles at 4 weeks each while hyperthermia will be administered for 1 hour on the same day as chemotherapy. Proton beam therapy is performed concurrently and the targeted dose is 67.5Gy

The TT-LAP trial includes safety evaluations in 5 initial patients as phase I (Fig. 2). After these initial 5 patients are enrolled, the last patient will complete multidisciplinary treatment followed by a 14-day observation period to assess adverse events (AEs) and severe AEs. If less than one case of DLT occurs within these 5 patients and safety is confirmed, the patients will be moved to the phase II efficacy evaluation. Judgment will be performed by an independent data monitoring committee.

### Definition of dose-limiting toxicity

Dose-limiting toxicity (DLT) is defined as an adverse event (AE) that is at least potentially related to the protocol treatment and meets the criteria. Each criterion is listed in Table 2. Grading of vitiligo or alopecia are not applicable to DLT. A DLT event will be assessed during the DLT evaluation period of this study, which will be 14 days from the start of protocol treatment to the end of treatment. DLT severity judgements will follow the guidelines described in Common Terminology Criteria for Adverse Events (CTCAE) ver. 5.0. If standard chemotherapy is continued during the DLT evaluation period, the patient will be considered to have discontinued the study and will not be included in the DLT evaluation. Additionally, if disease progression is observed during the DLT evaluation period, the study treatment will be discontinued and the patient will be excluded from the DLT evaluation.

**Table 2:**
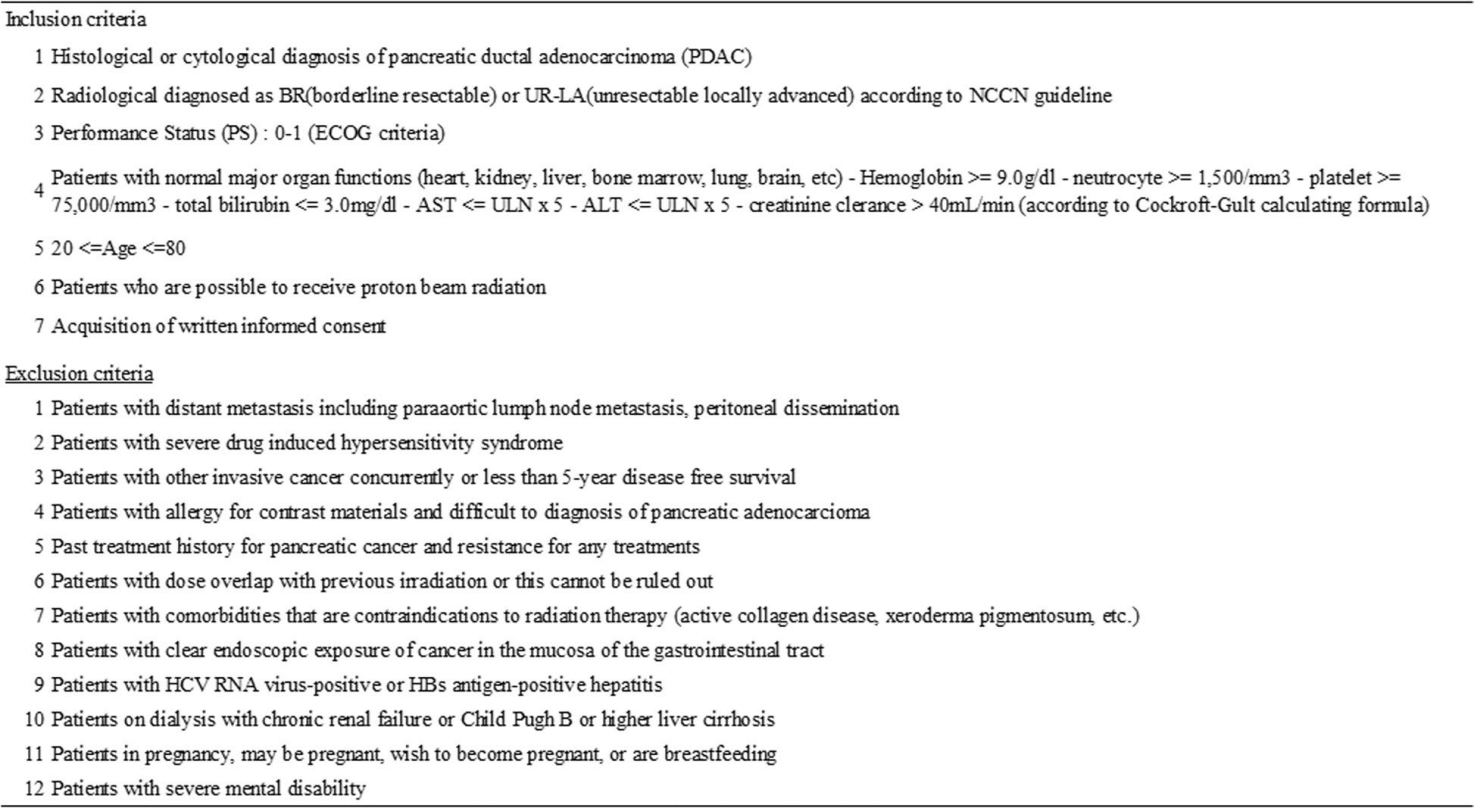
Inclusion and exclusion criteria of TT-LAP study

### Sample size determination

For the purpose of evaluating the safety of the study, the first 5 patients were assigned to Phase 1, which was a safety part of the study. The total number of patients in the study was set at 30 for feasibility and for the following reasons. The results of a single-center, retrospective, observational study on multimodality therapy using proton beams and hyperthermia with GEM alone [9] showed a 48% of 2-year survival rate for UR-LA pancreatic cancer. In addition, according to a randomized controlled trial of multidisciplinary treatment for locally advanced pancreatic cancer conducted in Japan (JCOG1106) [12], the 2-year survival rate of the CRT arm for locally advanced pancreatic cancer was 32%. Based on these results, thresholds and expected values were set as follows.

This number of cases meets the threshold response rate of 30% for 2-year survival when conventional chemoradiotherapy is administered, an expected response rate of 55%, and power set to 80% when the two-sided significance level is set at 0.05. This assumes a higher expected response rate to intensified chemotherapy and local hyperthermia than the 50% response rate for PBT and chemotherapy.

### Efficacy evaluations for phase 2

Efficacy will be evaluated by blood samples to measure tumor markers and contrast-enhanced CT scans of the thorax and abdomen, which will be scheduled every 3 months, to evaluate the presence of primary tumors and neoplastic lesions. For efficacy, overall survival will be analyzed by the Kaplan-Meier method to obtain a 2-year survival rate. The analysis of the primary efficacy endpoint will be based on the total analysis of the population (FAS).

### Trial resources

This study is funded by the Grant for Implementation of Advanced Medicine (GIAM) of the University of Tsukuba Hospital.

### Ethics and dissemination

This study was approved by the Tsukuba University Clinical Research Review Board based on the Clinical Trials Act of Japan and the Declaration of Helsinki or its equivalents. This trial is registered in the Japan Registry of Clinical Trials (jRCTs031220160). The results from this study will be analyzed and published in peer-reviewed journals.

## Discussion

### Rationale of the TT-LAP trial

Treatment of LAPC is extremely confounding since heterogenous disease progression creates unique treatment response profiles and high case-to-case variability. Compounding this difficulty is exact quantification of tumor and metastatic parameters only by currently available imaging studies. In addition, the high biological malignancy of pancreatic cancer cells and their resistance to chemotherapy and radiotherapy are also contributing factors. Thus, effective and recommended treatment strategies remain undefined. LAPC, in these cases, must be approached from the standpoint of not just solid tumors but also malignancies in active transition to micro-metastasis. Because of their multispectral targeting ability, intensive and synergistic chemotherapies (such as FOLFIRINOX) should therefore be considered as a mainstay for systemic treatment of LAPC since the previously reported median OS of 24.2 months is superior to gemcitabine for LAPC [7]. However, chemotherapy should, in principle, be continued permanently to suppress micro-metastases until radical resection can be achieved (if possible). Surgeons thus attempt to break this never-ending treatment cycle by ablation of the primary tumor even if radical resection cannot be achieved. Thus, multi-modal therapy buys crucial time and chances to achieve such resection by suppressing the rapid evolution of metastatic tumor cells as well as progression within the primary tumor. As in other combined therapy reports, our multimodality treatment results from clinical experience with the combination of PBT and single-agent gemcitabine, which has demonstrated a strong control effect and progression-free survival of more than 5 years in some patients [9]. Although we have seen cases with achievement of radical resection after triple-modal treatment (resulting in pathological complete response), control of frequent, often-invisible, and distant metastases after treatment require synergistic and systemic chemotherapy for both prophylaxis and treatment. From a standpoint of hematologic toxicity and adverse events, however, we concluded that gemcitabine/nab-paclitaxel (with milder side effects) is superior for our purposes versus FOLFIRINOX for such multidisciplinary treatments.

Hence, the aim of this clinical trial (TT-LAP) is to evaluate the safety and effectiveness of our triple-modal treatment, comprised of gemcitabine with nab-paclitaxel (standard for advanced PDAC), PBT, and local hyperthermia.

### Feasibility of treatment protocol

The TT-LAP trial includes an initial phase 1 safety assessment due to a lack of evidence when combining PBT with local hyperthermia and gemcitabine with nab-paclitaxel infusion for pancreatic cancer. In our previous report on gemcitabine-based, triple-modal treatment for LPAC [9], a gastric ulcer (grade 3, according to CACTE ver5.0) in 1 patient and neutropenia (grade 3 or 4) in 45% of enrolled patients were the primary adverse events, but no patient deaths or grade 4 adverse events were observed. Here, while we do expect gemcitabine with nab-paclitaxel to increase side effects due to hematotoxicity (and thus warrant a safety assessment), we chose it for the TT-LAP trial because it can be more safely introduced into multidisciplinary treatment in terms of adverse events [3, 6]. A similar ongoing clinical study, currently testing chemoradiotherapy in combination with conventional gemcitabine nab-paclitaxel therapy, employs 80% of the recommended dose and we mirrored that dose in this study.

### Advances in PBT

Several studies have reported the feasibility and effectiveness of PBT as the ideal radiation therapy for pancreatic cancer [9, 13–15]. However, to the best of our knowledge, there are no randomized clinical trials that have examined the therapeutic efficacy of photon versus proton beams. PBT, roughly equivalent in biological effect to X-rays, uses a particle accelerator to accelerate particles and harness their energy, packing 1,836 times more mass than photons. This unique characteristic gives them a Bragg peak that enables focusing at a specific body depth in the body to avoid excessive radiation doses to the surrounding organs [16]. Photon irradiation, conversely, dumps most of its total energy (integral dose) outside the target volume and increases the risk of adverse effects. For locally advanced pancreatic cancer, PBT carries the advantage of high on-target delivery to tumor bodies and the nerve plexus/feeder vessels while reducing the impact on the digestive tract. Our group has reported the efficacy of PBT for locally advanced pancreatic cancer and the survival of patients improved in a dose-dependent manner [15]. Furthermore, the 1-year/2-year survival rate was 83.3/78.9% with a median time-to-local-recurrence of more than 36 months. In univariate analyses, total irradiation dose was found to be the only significant factor for OS and local control. Several clinical trials reported to date also suggest that combinations of PBT and systemic chemotherapy for pancreatic cancer are extremely well tolerated and effective [13, 16]. Additionally, there is room and potential for further enhancement within each therapy to increase overall efficacy. Indeed, PBT was selected by the National Health Insurance of Japan as an insurable treatment option for unresectable, locally advanced pancreatic cancer starting from April 2022.

### Advances in hyperthermia

Hyperthermia has been widely applied to breast cancer and other malignant tumors of the body surface as well as to the multidisciplinary treatment of pancreatic cancer. A review by Horst et al. showed that hyperthermia can enhance the therapeutic effect of combinations of chemoradiotherapy or radiotherapy for pancreatic cancer [17]. In addition, no adverse events associated with hyperthermia have been reported, except for a grade 1 skin disorder in one case in which hyperthermia was administered intraoperatively. In our 21 recorded cases of adverse events, only localized erythema was observed with hyperthermia alone, thus confirming its safety. We have also experimentally demonstrated that hyperthermia increases drug accumulation in tumor cells in basic experiments [18].

### Differences from similar ongoing clinical trials

Clinical trials have been conducted for unresectable/regionally advanced pancreatic cancer using PBT in combination with chemotherapy to determine radiation dose [16]. Investigators at the University of Maryland, USA, are conducting a phase I and II clinical trial of gemcitabine nab-paclitaxel therapy with PBT (NCT03652428) similar to this study. The primary endpoint is to determine the maximum dose of PBT. In Japan, several centers conducted phase I/II trials of gemcitabine nab-paclitaxel combined with chemoradiotherapy for locally advanced pancreatic cancer [19, 20]. The primary endpoint of this study is also the 2-year survival rate, in order to show a comparison with these earlier studies [19, 20]. Our study differs in that hyperthermia is used in combination with the expectation of an additive synergistic effect. The safety of multidisciplinary chemoradiotherapy for pancreatic cancer should be verified through multiple validation cycles, including this study, while the efficacy should be verified in randomized controlled trials with chemotherapy alone.

## Conclusion

Although the number of facilities that can provide PBT and hyperthermia is limited, several trials have reported that PBT for locally advanced pancreatic cancer is well tolerated and has a high local control effect. A multimodality approach combining PBT, hyperthermia, and modern chemotherapy (TT-LAP trial) has the potential to achieve even stronger local control and control of micro-metastases.

## Fundings

The TT-LAP trial is funded by the Advanced Medical Care Promotion Support Program, University of Tsukuba Hospital (Reference Number: Senshin-99), which support the data management and monitoring of the trial.

## Data Availability

The full study protocol and all regulatory documents can be provided by Japan Registry of Clinical Trials (Trial ID: jRCTs031220160) https://jrct.niph.go.jp/en-latest-detail/jRCTs031220160.

## Abbreviations

AE: Adverse event
BR: Borderline Resectable
BR-A: Borderline Resectable with artery invasion
CT: Computed tomography
CTCAE: Common Terminology Criteria for Adverse Events
CR: Complete response
DLT: Drug-limiting toxicity
ECOG: Eastern Cooperative Oncology Group
FAS: Full set analysis
FDG-PET: 18F-fluorodeoxyglucose positron emission tomography
FOLFIRINOX: Combination chemotherapy regimen consisting of oxaliplatin, irinotecan, fluorouracil, and leucovorin
JPS: Japan Pancreas Society
jRCT: Japan Registry for Clinical Trials
MRI: Magnetic resonance imaging
LAPC: Locally Advanced Pancreatic Cancer
NCCN: National Comprehensive Cancer Network
CTCAE: Common Toxicity Criteria for Adverse Events
OS: Overall survival
PBT: Proton beam therapy
pCR: Pathological complete response
PDAC: Pancreatic ductal adenocarcinoma
PR: Partial response
PS: Performance status
RECIST: Response Evaluation Criteria In Solid Tumours
SAE: Serious adverse event
UR-M: Unresectable with distant metastasis
UR-LA: Unresectable locally advanced
